# Adaptive optimal control of under-actuated robotic systems using a self-regulating nonlinear weight-adjustment scheme: Formulation and experimental verification

**DOI:** 10.1371/journal.pone.0295153

**Published:** 2023-12-08

**Authors:** Omer Saleem, Mohsin Rizwan, Jamshed Iqbal

**Affiliations:** 1 Department of Electrical Engineering, National University of Computer and Emerging Sciences, Lahore, Pakistan; 2 Department of Mechatronics and Control Engineering, University of Engineering and Technology, Lahore, Pakistan; 3 School of Computer Science, Faculty of Science and Engineering, University of Hull, Hull, United Kingdom; University of Shanghai for Science and Technology, CHINA

## Abstract

This paper formulates an innovative model-free self-organizing weight adaptation that strengthens the robustness of a Linear Quadratic Regulator (LQR) for inverted pendulum-like mechatronic systems against perturbations and parametric uncertainties. The proposed control procedure is devised by using an online adaptation law to dynamically adjust the state weighting factors of LQR’s quadratic performance index via pre-calibrated state-error-dependent hyperbolic secant functions (HSFs). The updated state-weighting factors re-compute the optimal control problem to modify the state-compensator gains online. The novelty of the proposed article lies in adaptively adjusting the variation rates of the said HSFs via an auxiliary model-free online self-regulation law that uses dissipative and anti-dissipative terms to flexibly re-calibrate the nonlinear function’s waveforms as the state errors vary. This augmentation increases the controller’s design flexibility and enhances the system’s disturbance rejection capacity while economizing control energy expenditure under every operating condition. The proposed self-organizing LQR is analyzed via customized hardware-in-loop (HIL) experiments conducted on the Quanser’s single-link rotational inverted pendulum. As compared to the fixed-gain LQR, the proposed SR-EM-STC delivers an improvement of 52.2%, 16.4%, 55.2%, and 42.7% in the pendulum’s position regulation behavior, control energy expenditure, transient recovery duration, and peak overshoot, respectively. The experimental outcomes validate the superior robustness of the proposed scheme against exogenous disturbances.

## 1. Introduction

Under-actuated robotic systems are characterized as multivariable systems whose control inputs are less than the number of state variables or the system’s degrees of freedom (DOF) [1]. This control input limitation renders nonlinearity, complex dynamic coupling effects, difficulty in achieving multiple control objectives, and high susceptibility to exogenous disturbances [2]. The under-actuation property is observed in several mechanisms, such as inverted pendulums, self-stabilizing robots, aircraft and drones, satellites, robotic arm manipulators, and marine vessels [3]. The development of robust-optimal regulatory controllers for the under-actuated systems that can address the aforementioned challenges presents a complex control problem, as highlighted in [4].

### 1.1. Literature review

For the under-actuated systems, a lot of study and research has been done to develop versatile and reliable control methods. The proportional integral differential controllers offer simplicity and reliability, but they depend on well-postulated gains to attain the desired system specifications [5]. The fractional controllers increase the controller’s design flexibility by offering more tuning freedom [6]. However, tuning a multitude of controller parameters is an ill-posed problem. The conventional type-2 fuzzy control schemes offer flexibility in structure to compensate for bounded exogenous disturbances [7]. However, gathering elaborate rules to derive agile control decisions is a laborious process. The model-free neural control procedures offer robustness against bounded exogenous disturbances [8]. However, acquiring and processing large amounts of training data to formulate an accurate inverse control law is computationally expensive and time-consuming. The sliding-mode controllers are well known for their strong robustness against disturbances, which comes at the cost of a highly disputed control profile and, hence, may suffer from large chatter in the response [9]. The linear quadratic regulator (LQR) is an optimal control strategy that minimizes the quadratic cost function (QCF) of the system’s state variations and control input [10]. Despite its attributes, the LQR yields a fragile effort against modeling uncertainties, identification errors, and nonlinear disturbances [11]. Robust nonlinear H∞ controllers have also been widely used for the control of underactuated systems [12]. However, the boundary requirements and complex geometry of the system’s model impose limitations on computing its exact solution.

The inherent shortcomings of the generic LQR for an under-actuated mechatronic system can be alleviated by using online self-adaptive control mechanisms [13]. The adaptive control paradigm offers robust control effort by dynamically adjusting the critical controller parameters to eliminate the reference-tracking error and deviations in state trajectories of under-actuated systems that are caused by parametric uncertainties, bounded exogenous disturbances, and environmental indeterminacies [14, 15]. The model-reference adaptive systems are quite renowned for their agile control behavior [16]. However, deriving an accurate reference model to track and yield adaptive control decisions is a cumbersome process [17]. The nonlinear quadratic regulator (NQR) for under-actuated mechatronic systems can be systematically synthesized by utilizing state-dependent Riccati equation (SDRE) [18]. However, identifying accurate state-driven coefficients of the state-space matrices belonging to a higher-order nonlinear multivariable system (with complex geometry) is indeed a very difficult task.

Another self-adaptive LQR procedure that has recently gained a lot of attention works on the principle of self-tuning the weighting factors of its inner QCF [19]. The weighting matrices of LQR’s performance index put direct emphasis on the state variations and, thus, play a key role in dictating the optimal control profile for a given application [20]. The controller’s disturbance compensation capacity can be significantly enhanced if the weighting factors are adaptively configured online as a nonlinear scaling function of the system’s state error variables that are formulated as per pre-postulated meta-rules [21].

### 1.2. Proposed methodology

This article mainly contributes to synthesizing an adaptive LQR for under-actuated mechatronic systems that uses a novel self-organizing online self-tuning mechanism for the state weights in the QCF. The proposed control scheme is developed by retrofitting the nominal LQR with an online adaptation scheme that uses nonlinear scaling functions of state error variables to modify the state weights. To further increase the controller’s flexibility, the aforementioned weight adjustment scheme is retrofitted with a supplementary self-regulation mechanism that adaptively re-calibrates the variance of the nonlinear scaling functions. The standard single-link rotational pendulum (SRP) platform is used to experimentally analyze the efficacy of the prescribed control procedure in the physical environment. The three key contributions of this research are listed below:

1. Formulating customized hyperbolic secant functions (HSFs) that depend on the magnitudes of the classical state error and error-derivative variables to self-adjust each state weighting factor in the LQR’s internal QCF. The adjusted weights update the solution of optimal control problem to yield time-varying LQR gains.
2. Augmenting the aforementioned HSF-based adaptation law with a superior self-regulation mechanism that dynamically reconfigures the variation rates of each weight-adjusting HSF.
3. Hardware-in-loop (HIL) realization and validation of the proposed control procedure by carrying out reliable experiments on the Quanser QNET rotary pendulum board [22].

**Fig 1:**
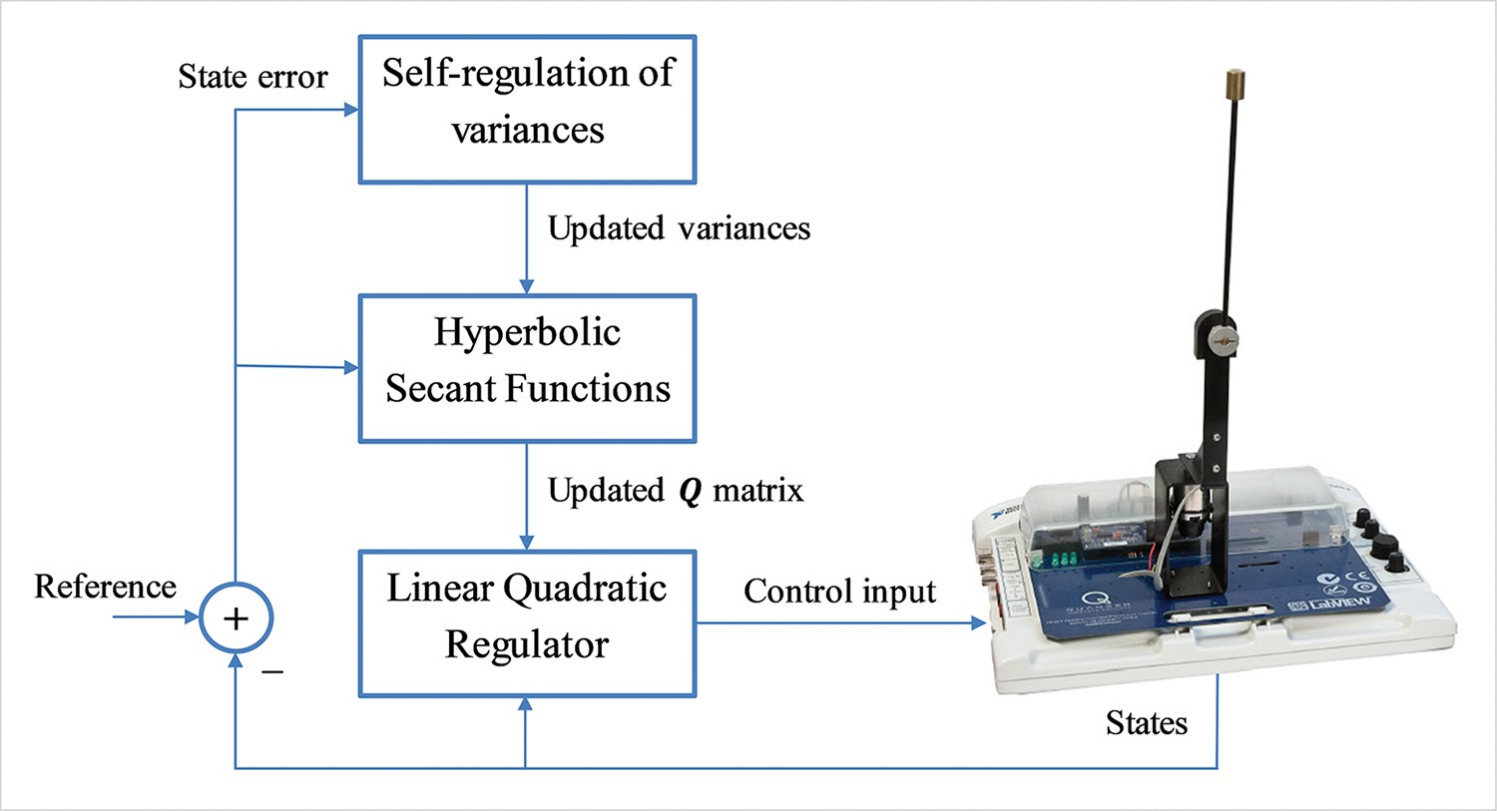
Schematic representation of the proposed control procedure.

The overall schematic of the proposed control procedure is shown in Fig 1.

### 1.3. Innovative features

The suggested control strategy offers several innovative features and advantages. The preset values of variation rates do not allow the adaptation functions to fully realize and handle the detrimental impacts of exogenous disturbances. Moreover, ill-calibrated variation rates normally lead to imprecise configuration of the scaling functions, which degrades the adaptability of the controller. The autonomous self-regulation of the functional variation rates, as proposed in this article, removes any residual inaccuracies rendered by the fixed tuning. The proposed self-regulation scheme uses dissipative and anti-dissipative blocks that capture the state error dynamics, which enhances the control scheme’s adaptability to efficiently react and apply appropriate control stiffness. This aids in yielding rapid transitions and strong damping against oscillations. This capability enables the adaptive system to execute accurate and efficient self-tuning of the variation rates. The self-regulation scheme is highly scalable as it does not rely on a priori information regarding the system’s mathematical model. The recursive computational burden imparted by the self-regulation scheme can be easily handled by the processing power of modern digital computers. As per the author’s knowledge at the time of writing this article, the aforementioned self-organizing adaptive LQR procedure has never been discussed in the available scientific literature. Hence, this article pivots around the hardware realization and verification of this innovative proposition.

The realization of the proposed control scheme relies upon the well-identified state-space model of the system as well as the accurate offline computation of the adaptation law parameters. This is indeed a cumbersome process. However, as validated later in the article (see Results and Analysis section), the benefits offered by the proposed scheme outweigh the aforesaid computational requirement(s).

The remainder of the article is structured as follows: The system’s mathematical model and the design of fixed-gain LQR are discussed in the SRP system description section. The adaptive LQR procedure and the associated self-organizing weight-adaptation scheme are contrived in the Proposed control methodology section. The results of HIL experiments are analyzed and discussed in the Results and Analysis section. A formal conclusion is presented in the Conclusion section.

## 2. SRP system description

**Fig 2:**
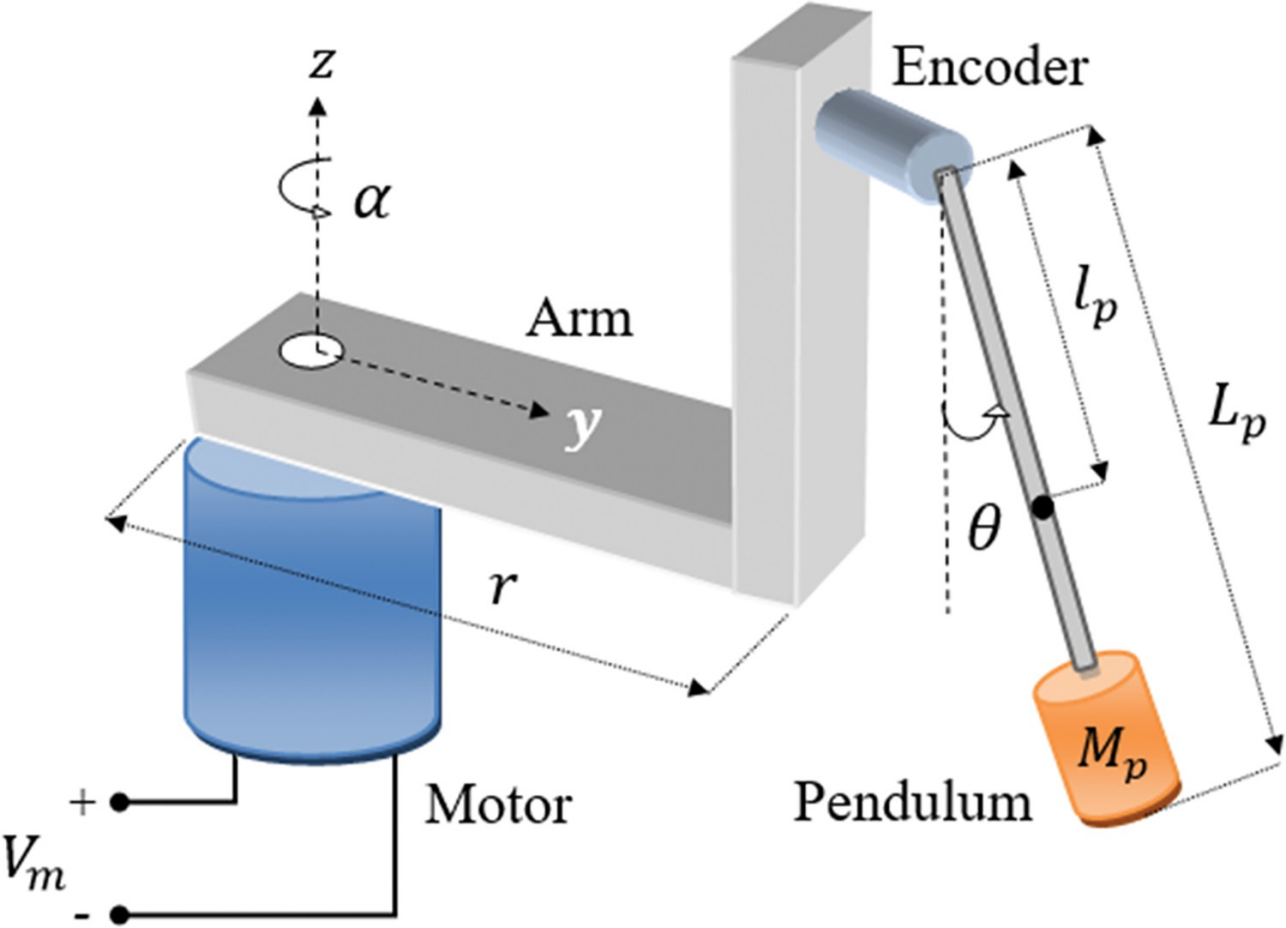
Schematic representation of a standard SRP system.

The SRP platform is widely favored for experimental verification and benchmarking of advanced control systems owing to its open-loop instability, under-actuated configuration, and nonlinear characteristics. Hence, in this article, the efficacy of the proposed adaptive LQR procedure is investigated via the SRP system. Fig 2 shows the hardware schematic for the standard SRP system.

The SRP system comprises a DC servo motor that rotates an arm hinged to its shaft. The rotation of the arm *α* is measured via the DC motor’s shaft encoder. The arm supports the pendulum rod assembly. The rotation of the arm energizes the rod to swing freely about the pivot point until it’s completely inverted. Once the rod has sufficient energy, it swings up and balances itself vertically. To track the rod’s angular displacement *θ*, a rotary encoder is mounted at its pivot. The SRP system is described in terms of *α* and *θ*, which are the arm’s and rod’s angular displacements, respectively.

### 2.1. Dynamic model of SRP system

The Euler-Lagrange method is used to derive the SRP system’s dynamic model [23]. The Lagrangian (*L*) is formulated as shown in (1).


$$L=E_{K}-E_{P}$$ (1)

$where,\;E_{P}=m_{p}l_{p}g(\cos\theta ),$

$and,\;E_{K}=\frac{1}{2}I_{e}{\left(\dot{\alpha }\right)}^{2}+\frac{1}{2}m_{p}{\left(r\dot{\alpha }-l_{p}\dot{\theta }(\cos\theta )\right)}^{2}+\frac{1}{2}m_{p}{\left(-l_{p}\dot{\theta }(\sin\theta )\right)}^{2}+\frac{1}{2}I_{p}{\left(\dot{\theta }\right)}^{2}$

The variables *E*_*P*_ and *E*_*K*_ represent the system’s total potential energy and total kinetic energy, respectively. The Lagrangian is formulated in (2), [24].


$$L=\frac{1}{2}(I_{e}+m_{p}r^{2}){\dot{\alpha }}^{2}+\frac{1}{2}(m_{p}{l_{p}}^{2}+I_{p}){\dot{\theta }}^{2}-m_{p}l_{p}r(\cos\theta )\dot{\alpha }\dot{\theta }-m_{p}l_{p}g(\cos\theta )$$ (2)

The system’s model parameters are described and identified in Table 1 [25]. The system’s equations of motion are derived via the expressions given in (3), [23].

$$\frac{\delta }{\delta t}(\frac{\delta L}{\delta \dot{\alpha }})-\frac{\delta L}{\delta \alpha }=\tau -b_{v}\dot{\alpha },\;\frac{\delta }{\delta t}(\frac{\delta L}{\delta \dot{\theta }})-\frac{\delta L}{\delta \theta }=0$$ (3)

where, *τ* is the control torque of the DC motor and *b*_*v*_ is the motor’s viscous damping.

**Table 1:** Model parameters of Quanser SRP.

Parameter	Description	Value	Unit
*I*_*e*_	Motor shaft’s moment	1.23×10^−4^	kgm^2^
*I*_*p*_	Pendulum rod’s moment	1.10×10^−4^	kgm^2^
*m*_*p*_	Pendulum rod’s mass	0.027	kg
*r*	Rotating arm’s length	0.083	m
*l*_*p*_	Pendulum center of mass	0.153	m
*L*_*p*_	Pendulum rod’s length	0.191	m
*m*_*arm*_	Rotating arm’s mass	0.028	kg
*g*	Gravitational acceleration	9.810	m/s^2^
*R*_*m*_	Motor’s resistance	3.30	Ω
*L*_*m*_	Motor’s inductance	47.0	mH
*K*_*t*_	Motor torque constant	0.028	Nm/A
*K*_*m*_	Back e.m.f. constant	0.028	V/(rad/s)
*τ*_*m*_	Maximum torque	0.14	Nm

It is ignored in the model due to its insignificant impact. The motor torque is expressed in (4).


$$\tau =\frac{K_{t}(V_{m}-K_{m}\dot{\alpha })}{R_{m}}$$ (4)

The motor’s control torque depends upon the input voltage *V*_*m*_. The solution of (3) yields the nonlinear Eqs given in (5) and (6) [25].


$$\ddot{\alpha }=\frac{-rm_{p}^{2}l_{p}^{2}g(\cos\theta )\theta -I_{p}m_{p}r^{2}\cos\theta \sin\theta {\left(\dot{\alpha }\right)}^{2}-(I_{p}+m_{p}l_{p}^{2})\tau }{(m_{p}r^{2}(\sin^{2}\theta )-I_{e}-m_{p}r^{2})I_{p}-m_{p}l_{p}^{2}I_{e}}$$ (5)

$$\ddot{\theta }=\frac{-m_{p}l_{p}((m_{p}r^{2}g(\sin^{2}\theta )-I_{e}g-m_{p}r^{2}g)\theta +rI_{e}\sin\theta {\left(\dot{\alpha }\right)}^{2}-r\;\tau \;\cos\theta )}{(m_{p}r^{2}(\sin^{2}\theta )-I_{e}-m_{p}r^{2})I_{p}-m_{p}l_{p}^{2}I_{e}}$$ (6)

The system linearization is done about the vertical position; where, $\alpha =\pi \;rad.,\;\theta =0,\;\dot{\alpha }=0,\;\dot{\theta }=0$. Furthermore, sin *θ*≈*θ* and cos *θ*≈1 are used to approximate the small-angle contributions. These approximations yield (7) and (8).

$$\ddot{\alpha }(t)=\frac{1}{G}\left(rm_{p}^{2}l_{p}^{2}g\theta (t)-\frac{(I_{p}+m_{p}l_{p}^{2})K_{t}K_{m}}{R_{m}}\dot{\alpha }(t)+\frac{(I_{p}+m_{p}l_{p}^{2})K_{t}}{R_{m}}V_{m}\right)$$ (7)

$$\ddot{\theta }(t)=\frac{1}{G}\left(m_{p}l_{p}g(I_{e}+m_{p}r^{2})\theta (t)-\frac{rm_{p}l_{p}K_{t}K_{m}}{R_{m}}\dot{\alpha }(t)+\frac{rm_{p}l_{p}K_{t}}{R_{m}}V_{m}\right)$$ (8)

$such\;that,\;G=I_{e}I_{p}+m_{p}r^{2}I_{p}+m_{p}l_{p}^{2}I_{e}$

The Eqs (7) and (8) are represented in state space form as expressed in (9).

$$\dot{x}(t)=Ax(t)+Bu(t),\;y(t)=Cx(t)+Du(t)$$ (9)

where, ***A*** is the system matrix, ***B*** is the input matrix, ***C*** is the output matrix, ***D*** is the feed-forward matrix, *u*(*t*) is the control input signal, *x*(*t*) is the state vector, and *y*(*t*) is the output vector. The system’s input vector and state vector are presented in (10).


$$u(t)=V_{m},\;x(t)={\left[\begin{array}{cc} \begin{array}{cc} \alpha (t) & \theta (t) \end{array} & \begin{array}{cc} \dot{\alpha }(t) & \dot{\theta }(t) \end{array} \end{array}\right]}^{T}$$ (10)

The SRP’s state-space model is provided in (11) [23].


$$A=[\begin{array}{cccc} 0 & 0 & 1 & 0\\ 0 & 0 & 0 & 1\\ 0 & a_{1} & a_{2} & 0\\ 0 & a_{3} & a_{4} & 0 \end{array}],\;B=[\begin{array}{c} 0\\ 0\\ b_{1}\\ b_{2} \end{array}],\;C=[\begin{array}{llll} 1 & 0 & 0 & 0\\ 0 & 1 & 0 & 0\\ 0 & 0 & 1 & 0\\ 0 & 0 & 0 & 1 \end{array}],\;D=[\begin{array}{l} 0\\ 0\\ 0\\ 0 \end{array}]$$ (11)

$$where,\;a_{1}=\frac{rM_{p}^{2}l_{p}^{2}g}{J_{p}J_{e}+J_{e}l_{p}^{2}M_{p}+J_{p}M_{p}r^{2}},\;a_{2}=\frac{-K_{t}K_{m}(J_{p}+M_{p}l_{p}^{2})}{(J_{p}J_{e}+J_{e}l_{p}^{2}M_{p}+J_{p}M_{p}r^{2})R_{m}},$$

$$a_{3}=\frac{M_{p}l_{p}g(J_{e}+M_{p}r^{2})}{J_{p}J_{e}+J_{e}l_{p}^{2}M_{p}+J_{p}M_{p}r^{2}},\;a_{4}=\frac{-rM_{p}l_{p}K_{t}K_{m}}{(J_{p}J_{e}+J_{e}l_{p}^{2}M_{p}+J_{p}M_{p}r^{2})R_{m}},$$

$$b_{1}=\frac{K_{t}(J_{p}+M_{p}l_{p}^{2})}{(J_{p}J_{e}+J_{e}l_{p}^{2}M_{p}+J_{p}M_{p}r^{2})R_{m}},\;b_{2}=\frac{rM_{p}l_{p}K_{t}}{(J_{p}J_{e}+J_{e}l_{p}^{2}M_{p}+J_{p}M_{p}r^{2})R_{m}}$$

### 2.2. Baseline linear control scheme

The ubiquitous LQR is a state compensator that employs the full state feedback of a linear system by delivering optimal regulatory control input [19]. It achieves the said optimality by minimizing the QCF provided in (12) [11].

$$J_{lq}=\frac{1}{2}\int_{0}^{\infty }\left({x(t)}^{T}Qx(t)+{u(t)}^{T}Ru(t)\right)dt$$ (12)

where, ***R***∈ℝ is a positive definite control input weighting matrix and ***Q***∈ℝ^4×4^ is a positive semi-definite state weighting matrix. The Hamilton-Jacobi-Bellman (HJB) equations are then used to evaluate the state compensator gains. The ***R*** and ***Q*** matrices associated with the standard SRP system are denoted in (13).

$$R=\rho ,\;Q=diag(\begin{array}{ccc} q_{\alpha } & q_{\theta } & \begin{array}{cc} q_{\dot{\alpha }} & q_{\dot{\theta }} \end{array} \end{array})$$ (13)

where, *ρ*>0 and *q*_*x*_≥0 are the predetermined constituent factors of the ***R*** and ***Q*** matrices respectively. The offline optimization of these matrices is discussed in the Parameter tuning procedure section. The tuned ***R*** and ***Q*** matrices are utilized to solve the Algebraic-Riccati-Equation (ARE), shown in (14), and evaluate the ***P*** matrix.

$$A^{T}P+PA-PBR^{-1}B^{T}P+Q=0$$ (14)

where, ***P***∈ℝ^4×4^ is a positive definite matrix. The state-compensator gain vector (***K***) for the SRP system is calculated as shown in (15).

$$K=R^{-1}B^{T}P$$ (15)

where, $K=[\begin{array}{cc} \begin{array}{cc} k_{\alpha } & k_{\theta } \end{array} & \begin{array}{cc} k_{\dot{\alpha }} & k_{\dot{\theta }} \end{array} \end{array}]$. The fixed-gain LQR law is presented in (16).


u(t)=−Kx(t) (16)

The LQR’s Lyapunov stability is verified via the following function.


$$V(t)={x(t)}^{T}Px(t)>0,\;for\;x(t)\neq 0$$ (17)

The derivative of *V*(*t*) is derived in (18).


$$\dot{V}(t)=2{x(t)}^{T}P\dot{x}(t)$$ (18)

$$=2{x(t)}^{T}P(A-BK)x(t)$$

$$=2{x(t)}^{T}P(A-BR^{-1}B^{T}P)x(t)$$

$$={x(t)}^{T}(PA+A^{T}P)x(t)-2{x(t)}^{T}(PBR^{-1}B^{T}P)x(t)$$

By making necessary substitutions from (18), $\dot{V}(t)$ simplified as expressed in (19).


$$\dot{V}(t)=-{x(t)}^{T}Qx(t)-{x(t)}^{T}(PBR^{-1}B^{T}P)x(t)\;<0$$ (19)

The function $\dot{V}(t)$ is negative-definite if ***R*** = ***R***^*T*^>0 and ***Q*** = ***Q***^*T*^≥0. These conditions are adequate to maintain the LQR’s asymptotic stability. The LQR law is robustified by supplementing it with integral-of-error control terms as shown in (20).

$$u_{i}(t)=K_{i}\varepsilon (t)=[\begin{array}{cc} K_{i\alpha } & K_{i\theta } \end{array}]\left[\begin{array}{c} \varepsilon_{\alpha }(t]\\ \varepsilon_{\theta }(t) \end{array}\right]$$ (20)

where, ***K***_*i*_ is the integral gain vector, and *ε*_*α*_(*t*) and *ε*_*θ*_(*t*) are the integral-of-error variables given in (21)

$$\varepsilon_{\alpha }(t)=\int_{0}^{t}e_{\alpha }(\tau )d\tau ,\;\varepsilon_{\theta }(t)=\int_{0}^{t}e_{\theta }(\tau )d\tau$$ (21)

$such\;that,\;e_{\alpha }(t)=\alpha (0)-\alpha (t),\;e_{\theta }(t)=\pi -\theta (t)$ where, *e*_*α*_(*t*) and *e*_*θ*_(*t*) are the state-regulation errors linked with the pendulum’s arm and the rod, respectively. This modification improves the SRP’s balancing control behavior and increases damping against fluctuations [20]. The linear control scheme is restructured as shown in (22).


$$u(t)=-Kx(t)+K_{i}\varepsilon (t)$$ (22)

### 2.3. Parameter tuning procedure

The selection of the coefficients of the ***R*** and ***Q*** matrices is indeed a difficult task. For a specific set of ***R*** and ***Q*** matrices, the LQR gains do not necessarily offer accurate reference-tracking behavior and economical control activity simultaneously and, thus, a trade-off is generally made [21]. The control input weight (*ρ*) and the ***Q*** matrix are tuned by minimizing the objective function, expressed in (23), which minimizes the state errors and the input signal with equal weights [20].


$$J_{c}=\int_{0}^{\infty }\left({\left|e_{\alpha }(t)\right|}^{2}+{\left|e_{\theta }(t)\right|}^{2}+{\left|V_{m}(t)\right|}^{2}\right)dt$$ (23)

**Fig 3:**
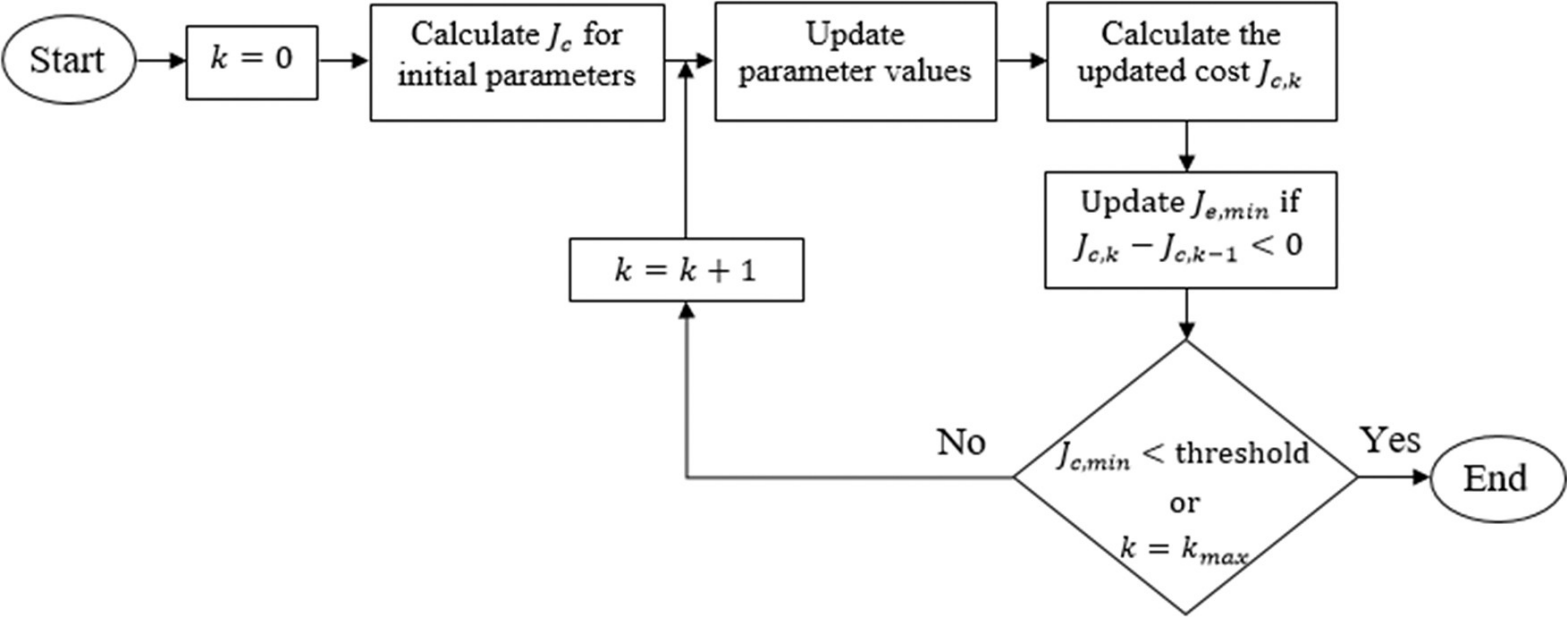
Flow chart of the parameter tuning procedure.

The objective function applies equal weight (unity) to each minimization criterion. The coefficients of ***Q*** matrix are selected from the range [0, 100], and the control weighting coefficient is selected from the range [0, 2]. The flow chart of the parameter tuning procedure is illustrated in Fig 3. The initial values of these parameters can be selected randomly from the afore-mentioned search space, and the algorithm then handles the exploration in the direction of the steepest gradient descent. Hence, keeping in view the lower bounds on the state and control weighting parameters, the tuning process is begun with $Q=diag(\begin{array}{ccc} 1 & 1 & \begin{array}{cc} 1 & 1 \end{array} \end{array})$ and ***R*** = 1 in this work. The tuning is conducted via a series of experimental trials under nominal conditions. The experimental procedure is discussed in the Hardware-in-the-loop experiments section. In every trial, the parameters are updated appropriately, the SRP’s rod is manually erected and balanced for 10.0 seconds to compute the resulting cost *J*_*c*,*k*_ for that trial, where *k* is the trial number. The tuning algorithm explores the range space in the direction of the plunging gradient of *J*_*c*_. If the cost of the current trial (*J*_*c*,*k*_) turns out to be less than the cost of the previous trial (*J*_*c*,*k*−1_), the local minimum-cost variable *J*_*c*,*min*_ is updated. The tuning process is concluded if either the algorithm has completed the maximum number of trials allowed or *J*_*c*,*min*_ has achieved the predefined threshold value. In this research, the predefined threshold for *J*_*c*,*min*_ is set at 1.0×10^4^, and the maximum number of trials (*k*_*max*_) allowed is 30. These settings are deduced as per the expert’s experience. The matrices thus obtained are $R=1.02,\;Q=diag(\begin{array}{ccc} 32.8 & 52.2 & \begin{array}{cc} 6.1 & 2.5 \end{array} \end{array})$. The conveyance of the optimized ***R*** and ***Q*** matrices to the ARE yields the gain vector, $K=[\begin{array}{ccc} -6.21 & 130.56 & \begin{array}{cc} -4.22 & 17.83 \end{array} \end{array}]$. The aforementioned procedure is also used to tune the integral gains in the range [–5, 0]. The integral gain vector thus obtained is $K_{i}=[\begin{array}{cc} -2.06 & -7.47\times 10^{-6} \end{array}]$.

## 3. Proposed control methodology

As discussed earlier, the weighting coefficients are chosen such that *ρ*>0 and *q*_*x*_≥0. The SRP system’s DOFs are greater than the rank of the ***R*** matrix, which verifies its under-actuation configuration. Hence, it is quite challenging to address all state error variables using a single control input. On the contrary, the state weighting coefficients (*q*_*x*_) maintain a one-to-one correlation with the corresponding state variables. Hence, in this research, the value of *ρ* is preset at 1.02 (as prescribed in the Parameter tuning procedure section), while the values of *q*_*x*_ are self-adjusted online via an adaptation scheme that effectively manipulates the control input trajectory to achieve the desired control objectives.

The proposed weight-adjustment scheme is devised via nonlinear scaling functions that capture the magnitudes of the state errors and their corresponding derivatives. The waveform and shape of the said functions are calibrated offline via the tuning procedure discussed in the Parameter tuning procedure section. The expression of the time-varying error-dependent weighting matrices is given in (24).


$$R=1.02,\;Q(t)=diag(\begin{array}{ccc} q_{\alpha }(e_{\alpha },t) & q_{\theta }(e_{\theta },t) & \begin{array}{cc} q_{\dot{\alpha }}({\dot{e}}_{\alpha },t) & q_{\dot{\theta }}({\dot{e}}_{\theta },t)) \end{array} \end{array}$$ (24)

The prescribed ***R*** matrix and the updated ***Q***(*t*) matrix are used to re-compute the Riccati equation’s updated solution ***P***(*t*), as shown in (25), after every sampling interval.


$$A^{T}P(t)+P(t)A-P(t)BR^{-1}B^{T}P(t)+Q(t)=0$$ (25)

The matrix ***P***(*t*) thus delivers the self-adjusting gain vector ***K***(*t*), as shown in (26).


$$K(t)=R^{-1}B^{T}P(t)$$ (26)

The self-adaptive LQR scheme is redefined in (27).


$$u(t)=-K(t)x(t)+K_{i}\varepsilon (t)$$ (27)

In the research, the vector ***K***(*t*) is altered via the proposed technique while the vector ***K***_*i*_ is affixed to the prescribed values. As discussed earlier, the ARE’s solution ensures the adaptive LQR’s asymptotic stability if $Q(t)={Q(t)}^{T}\geq 0$ and ***R*** = ***R***^*T*^>0. The online weight adjustment functions are customized to make sure that the values of *q*_*x*_ are always positive and semi-definite. The formulation of the tow weight adjustment mechanisms is presented as follows.

### 3.1. Basic weight adjustment scheme

The state weights of QCF are self-tuned online using well-configured scaling functions of the magnitudes of state error and error derivative variables. The following qualitative rules dictate the self-adaptation procedure for the weighting factors [26].

1. The weights *q*_*α*_ and *q*_*θ*_ are amplified as the magnitudes of state errors increase, and vice versa.
2. The weights $q_{\dot{\alpha }}$ and $q_{\dot{\theta }}$ are reduced as the magnitudes of error derivatives increase, and vice versa.

The aforementioned rationale renders flexible self-adaptability in the control scheme necessary to achieve the desired control objectives [27, 28]. It enhances the response speed, strengthens the damping against exogenous disturbances, quickly attenuates the overshoots or undershoots, and reduces the peak actuating torques. In this work, the weights are dynamically adjusted via pre-configured HSFs that comply with the aforementioned rationale. The HSFs are smooth, even-symmetric, and bounded between 0 and 1. They do not require a priori knowledge regarding the system’s model and can be easily formulated based on the expert’s experience. The generalized weight-adjusting function used in this research is expressed in (28).

$$q_{x}(e_{x},t)=a_{x}\pm b_{x}\mathrm{sech}\left(\gamma_{x}^{*}{\left|e_{x}(t)\right)}^{\varphi_{x}}\right)$$ (28)

where, *a*_*x*_ and *b*_*x*_ are used to decide the lower and upper limits of the weighting factor, respectively, sech(.) denotes the HSF, $\gamma_{x}^{*}$ is the weight adjusting function’s variation rate, *e*_*x*_(*t*) is the generalized state error variable, and *φ*_*x*_ is the fractional exponent of the error variable. The values of *a*_*x*_ and *b*_*x*_ are selected such that the weighting factors are always greater than zero to maintain the system’s closed-loop stability. These functions ensure the smooth commutation of state weights across all operating conditions. The weight-adjusting functions, designed to address each state-weighting factor, is expressed in (29) to (32).


$$q_{\alpha }(e_{\alpha },t)=a_{\alpha }+b_{\alpha }\mathrm{sech}\left(\gamma_{\alpha }^{*}.{\left|e_{\alpha }(t)\right|}^{\varphi_{\alpha }}\right)$$ (29)

$$q_{\theta }(e_{\theta },t)=a_{\theta }+b_{\theta }\mathrm{sech}\left(\gamma_{\theta }^{*}.{\left|e_{\theta }(t)\right|}^{\varphi_{\theta }}\right)$$ (30)

$$q_{\dot{\alpha }}({\dot{e}}_{\alpha },t)=a_{\dot{\alpha }}-b_{\dot{\alpha }}\mathrm{sech}\left(\gamma_{\dot{\alpha }}^{*}.{\left|{\dot{e}}_{\alpha }(t)\right|}^{\varphi_{\dot{\alpha }}}\right)$$ (31)

$$q_{\dot{\theta }}({\dot{e}}_{\theta },t)=a_{\dot{\theta }}-b_{\dot{\theta }}\mathrm{sech}\left(\gamma_{\dot{\theta }}^{*}.{\left|{\dot{e}}_{\theta }(t)\right|}^{\varphi_{\dot{\theta }}}\right)$$ (32)

**Fig 4:**
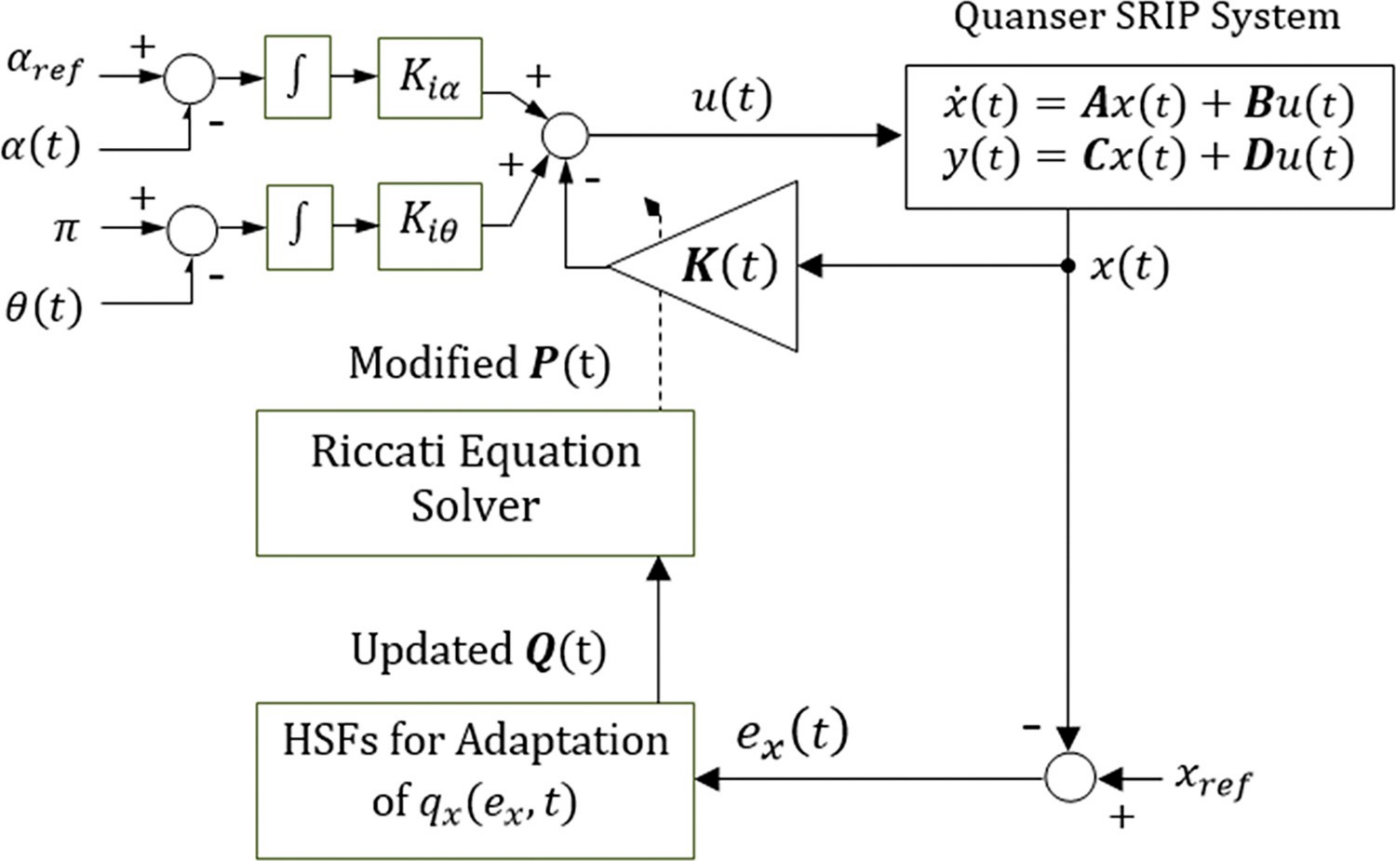
Block diagram of the baseline EM–STC scheme.

The hyper-parameters of each function are empirically calibrated offline via the procedure discussed earlier to attain a fast response speed with minimum regulation errors. The variation rate and the fractional exponent of each state error variable are selected from the range [0, 10]. A larger bandwidth results in abrupt variations in state weights that eventually lead to discontinuous control signal generation and inject chattering into the response. A smaller bandwidth of variation range renders the functions unable to quickly address rapidly changing error conditions. The upper and lower bounds are selected from the range [0, 500] to allocate adequate control resources as the error conditions change. This bandwidth was chosen as per the findings reported in [27]. The initial value of each parameter is set to unity for the tuning procedure. The resulting selected values of the parameters are given as; *a*_*α*_ = 1.85, *a*_*θ*_ = 2.41, $a_{\dot{\alpha }}$ = 11.05, $a_{\dot{\theta }}$ = 10.05, *b*_*α*_ = 408.15, *b*_*θ*_ = 291.62, $b_{\dot{\alpha }}$ = 10.58, $b_{\dot{\theta }}$ = 9.92, $\gamma_{\alpha }^{*}$ = 2.10, $\gamma_{\theta }^{*}$ = 5.76, $\gamma_{\dot{\alpha }}^{*}$ = 1.05, $\gamma_{\dot{\theta }}^{*}$ = 2.94, *φ*_*α*_ = 1.52, *φ*_*θ*_ = 1.45, $\varphi_{\dot{\alpha }}$ = 1.67, and $\varphi_{\dot{\theta }}$ = 1.62. Although this scheme introduces a multitude of hyper-parameters, which makes the tuning procedure, the performance-related benefits offered by the scheme surpass this drawback. The adaptive LQR augmented with the adaptation scheme presented in (29) to (32) is denoted as the Error Magnitude-driven Self Tuning Controller (or EM-STC) in this article. Fig 4 depicts the block diagram of the basic EM-STC.

### 3.2. Proposed self-organizing weight-adjustment scheme

Selecting fixed variation rates to optimize the shape and form of the aforesaid nonlinear weight-adjusting functions is an ill-posed problem. The fixed variation rates limit the controller’s disturbance rejection ability and incapacitate it to handle parametric variations and environmental indeterminacies. This inefficacy can be alleviated by autonomously self-regulating the variation rate of each weight-adjusting function via a superior self-regulator. This modification dynamically adjusts the rate of inflation or depreciation of the state weights, which improves the sensitivity and responsiveness of the adaptation scheme to handle rapid state error variations. Consequently, the adaptation scheme can flexibly drive the softness (or stiffness) of the applied control force, which efficiently compensates for the exogenous disturbances in minimal time with minimal oscillations and minimal control energy expenditure. Moreover, it also removes any shortcomings in the heuristic calibration of the weight-adjusting functions.

In this research, the variation rates are adapted online by using the online self-regulation law, prescribed by Fisher et al. in [29], due to its robust and accurate tracking ability. This online adaptation mechanism employs pre-calibrated dissipative and anti-dissipative functions that dynamically manipulate the variation rates based on the state error and error-derivative variations after every sampling interval. The time-varying self-regulating variance (SRV) functions are formulated in (33) to (36) [20, 30].

$$\dot{g_{\alpha }}(t)=-\sigma_{\alpha }g_{\alpha }(t)+\beta_{\alpha }e_{\alpha }^{2}(t)$$ (33)

$$\dot{g_{\theta }}(t)=-\sigma_{\theta }g_{\theta }(t)+\beta_{\theta }e_{\theta }^{2}(t)$$ (34)

$$\dot{g_{\dot{\alpha }}}(t)=-\sigma_{\dot{\alpha }}g_{\dot{\alpha }}(t)+\beta_{\dot{\alpha }}e_{\alpha }(t){\dot{e}}_{\alpha }(t)$$ (35)

$$\dot{g_{\dot{\theta }}}(t)=-\sigma_{\dot{\theta }}g_{\dot{\theta }}(t)+\beta_{\dot{\theta }}e_{\theta }(t){\dot{e}}_{\theta }(t)$$ (36)

where *g*_*x*_(*t*) represents the online self-regulation law that dynamically adjusts the variation rates by incrementing (or decrementing) their values, as shown in (37).

$$\gamma_{x}^{tot}(t)=\gamma_{x}^{*}+g_{x}(t)$$ (37)

where $\gamma_{x}^{tot}(t)$ is the final (adjusted) value of the variation rate and $\gamma_{x}^{*}$ is the nominal value of the variation rate (prescribed in the Basic weight adjustment scheme section). These aforementioned SRV functions alter the system’s responsiveness and tightness of control effort as the system deviates from or settles at the desired set point [30]. The parameters *β*_*x*_ and *σ*_*x*_ in (33) to (36) are the predefined positive adaptation rates and damping rates linked with each SRV function, respectively. These rates are optimized via the procedure discussed earlier. The selection range for *σ*_*x*_ is restricted to [0, 1] to allow for a gradual dissipative operation, while the selection range for *β*_*x*_ is restricted to [0, 10] to ensure a responsive anti-dissipative operation. The initial value of the decay rates is set to 0.01 and that of the adaptation rates is set to unity for the tuning procedure. The consequent selected values of these rates are given as; *σ*_*α*_ = 0.036, *σ*_*θ*_ = 0.045, $\sigma_{\dot{\alpha }}$ = 0.018, $\sigma_{\dot{\theta }}$ = 0.024, *β*_*α*_ = 0.65, *β*_*θ*_ = 0.88, $\beta_{\dot{\alpha }}$ = 5.82, and $\beta_{\dot{\theta }}$ = 7.05. The online adaptation begins with the initial values of *g*_*x*_(*t*), and after every sampling interval, the corresponding changes in their value are used to alter the variation rates as a function of the state errors. Each SRV function comprises a dissipative and an anti-dissipative term, as shown below [20].

$$Dissipative\;term:\;\left\{ \begin{array}{c} -\sigma_{\alpha }g_{\alpha }(t)\\ -\sigma_{\theta }g_{\theta }(t)\\ -\sigma_{\dot{\alpha }}g_{\dot{\alpha }}(t)\\ -\sigma_{\dot{\theta }}g_{\dot{\theta }}(t) \end{array}\right.\;Anti‐dissipative\;term:\;\left\{ \begin{array}{c} \beta_{\alpha }e_{\alpha }^{2}(t)\\ \beta_{\theta }e_{\theta }^{2}(t)\\ \beta_{\dot{\alpha }}e_{\alpha }(t){\dot{e}}_{\alpha }(t)\\ \beta_{\dot{\theta }}e_{\theta }(t){\dot{e}}_{\theta }(t) \end{array}\right.$$

The anti-dissipative terms enlarge the corresponding variance as the state error increases, and vice versa. This helps to apply a tighter control to dampen the overshoots in minimum time. The dissipative term slows the rate-of-change of the corresponding variance exponentially under low error conditions when the system is in the equilibrium state or when the anti-dissipative term becomes less dominant. This helps to attenuate steady-state perturbations, minimize the control energy consumption, and prevent wind-up. The four SRV functions are unified and represented as a first-order differential Eq in (38).

$$\dot{G}(t)=-FG(t)+Hv(t)$$ (38)

$$\mathrm{such}\;\mathrm{that},\;G(t)=[\begin{array}{l} g_{\alpha }(t)\\ g_{\theta }(t)\\ g_{\dot{\alpha }}(t)\\ g_{\dot{\theta }}(t) \end{array}],\;F=[\begin{array}{cccc} \sigma_{\alpha } & 0 & 0 & 0\\ 0 & \sigma_{\theta } & 0 & 0\\ 0 & 0 & \sigma_{\dot{\alpha }} & 0\\ 0 & 0 & 0 & \sigma_{\dot{\theta }} \end{array}],\;H=[\begin{array}{cccc} \beta_{\alpha } & 0 & 0 & 0\\ 0 & \beta_{\theta } & 0 & 0\\ 0 & 0 & \beta_{\dot{\alpha }} & 0\\ 0 & 0 & 0 & \beta_{\dot{\theta }} \end{array}],\;v(t)=[\begin{array}{c} e_{\alpha }^{2}(t)\\ e_{\theta }^{2}(t)\\ e_{\alpha }(t){\dot{e}}_{\alpha }(t)\\ e_{\theta }(t){\dot{e}}_{\theta }(t) \end{array}]$$

where *G*(*t*) is a vector containing the gains of the time-varying variation rates, *v*(*t*) is the vector of error-dependent terms, and the matrices ***F*** and ***H*** are positive-definite matrices containing the damping rates *σ*_*x*_ and the adaptation rates *β*_*x*_, respectively. After every sample period, the differential Eq in (38) is numerically integrated to acquire the *G*(*t*) and, hence, the updated values of the variation rates. The adaptation scheme is computationally realized by solving the differential Eq in (38) as shown in (39).

$$G(t)=exp(-Ft)G(0)+\int_{0}^{t}\left(exp(-F(t-p))Hv(p)\right)dp$$ (39)

where *exp*(.) represents the exponential function. The initial vector $G(0)={\left[\begin{array}{ccc} 0 & 0 & \begin{array}{cc} 0 & 0 \end{array} \end{array}\right]}^{T}$. The updated values of *g*_*x*_(*t*) are subsequently used to modify the variation rates as given in (40) to (43).


$$\gamma_{\alpha }^{tot}(t)=\gamma_{\alpha }^{*}+g_{\alpha }(t)$$ (40)

$$\gamma_{\theta }^{tot}(t)=\gamma_{\theta }^{*}+g_{\theta }(t)$$ (41)

$$\gamma_{\dot{\alpha }}^{tot}(t)=\gamma_{\dot{\alpha }}^{*}+g_{\dot{\alpha }}(t)$$ (42)

$$\gamma_{\dot{\theta }}^{tot}(t)=\gamma_{\dot{\theta }}^{*}+g_{\dot{\theta }}(t)$$ (43)

Finally, the changes in the updated values of $\gamma_{x}^{tot}(t)$ are bounded within ±*P*% of the nominal variation rate via a saturation function. This restriction prevents large overshoots, disrupted control activity, chattering in the response, and wind-ups. The saturation function is formulated in (44).

$$\gamma_{x}^{sat}(t)=\left\{ \begin{array}{l} (1+0.01P)\gamma_{x}^{*},\;\gamma_{x}^{tot}(t)\geq (1+0.01P)\gamma_{x}^{*}\\ \gamma_{x}^{tot}(t),\;(1-0.01P)\gamma_{x}^{*}<\gamma_{x}^{tot}(t)<(1+0.01P)\gamma_{x}^{*}\\ (1-0.01P)\gamma_{x}^{*},\;\gamma_{x}^{tot}(t)\leq (1-0.01P)\gamma_{x}^{*} \end{array}\right.$$ (44)

where $\gamma_{x}^{sat}(t)$ represents the saturated value of the adjustable variation rate. To improve the position-regulation accuracy and economize the control effort, the value of *P* is empirically set at 80.0 via trial-and-error. The updated formulae of the weight-adjusting functions are expressed in (45) to (48).


$$q_{\alpha }(e_{\alpha },t)=a_{\alpha }+b_{\alpha }\mathrm{sech}\left(\gamma_{\alpha }^{sat}(t).{\left|e_{\alpha }(t)\right|}^{\varphi_{\alpha }}\right)$$ (45)

$$q_{\theta }(e_{\theta },t)=a_{\theta }+b_{\theta }\mathrm{sech}\left(\gamma_{\theta }^{sat}(t).{\left|e_{\theta }(t)\right|}^{\varphi_{\theta }}\right)$$ (46)

$$q_{\dot{\alpha }}({\dot{e}}_{\alpha },t)=a_{\dot{\alpha }}-b_{\dot{\alpha }}\mathrm{sech}\left(\gamma_{\dot{\alpha }}^{sat}(t).{\left|{\dot{e}}_{\alpha }(t)\right|}^{\varphi_{\dot{\alpha }}}\right)$$ (47)

$$q_{\dot{\theta }}({\dot{e}}_{\theta },t)=a_{\dot{\theta }}-b_{\dot{\theta }}\mathrm{sech}\left(\gamma_{\dot{\theta }}^{sat}(t).{\left|{\dot{e}}_{\theta }(t)\right|}^{\varphi_{\dot{\theta }}}\right)$$ (48)

**Fig 5:**
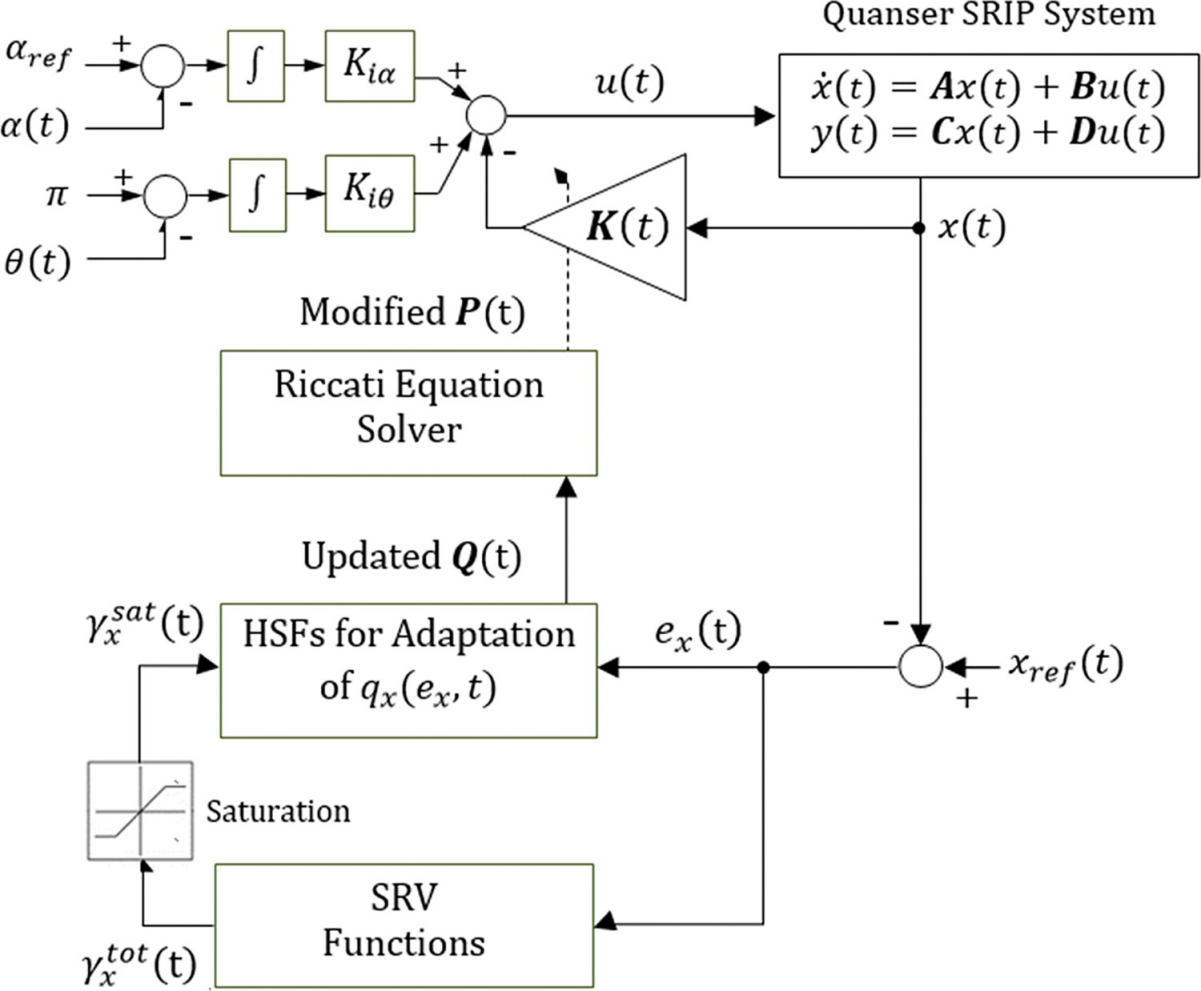
Block diagram of the proposed SR–EM–STC procedure.

The modified weight adjusting functions utilize the same prescribed values of *a*_*x*_, *b*_*x*_, and *φ*_*x*_ that were selected in the Basic weight adjustment scheme section, and only the variation rates $\gamma_{x}^{sat}(t)$ are being dynamically adjusted. The procedure for computing the time-varying state compensator gains is the same as prescribed in (26). The updated variation rates are fed to the weight-adjusting functions that alter the state-weighting factors to dynamically re-adjust the Riccati equation’s solution and yield the self-adjusting LQR gain vector ***K***(*t*). The adaptive LQR scheme augmented with the SRV functions is denoted as self-regulating EM-STC (or SR-EM-STC) in the remaining article. Fig 5 illustrates the proposed SR-EM-STC block diagram.

## 4. Results and analysis

This section comprehensively discusses the experimentation procedure for the HIL realization of the devised control strategies using the Quanser SRP setup.

### 4.1. Hardware platform

**Fig 6:**
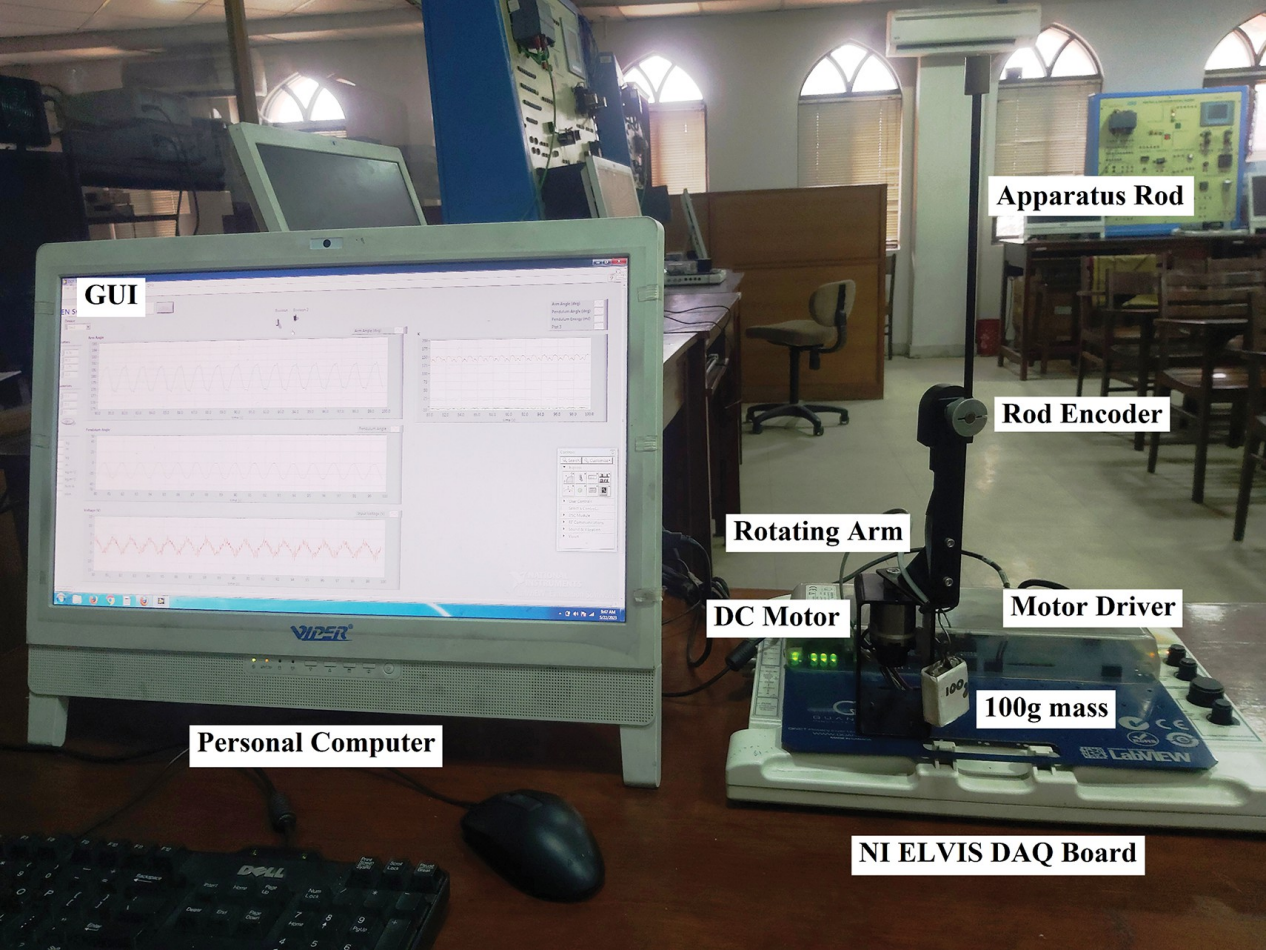
Quanser SRP setup used for experiments.

The robustness of the suggested controller variants is examined by performing HIL experiments on the QNET SRP platform, as depicted in Fig 6.

The real-time measurements of the angular positions of SRP’s arm and rod are acquired (from the respective encoders) at a sampling frequency of 1.0 kHz by using the NI DAQ Board. The acquired data is filtered and transmitted to the control software application over a serial communication link of 9600 bps. The personalized control application is tailored by using the "Block Diagram" tool of the LabVIEW software. A 64-bit, 2.1 GHz Intel Core i7 embedded personal computer with 16.0 GB of RAM is used to run the control software. The hardware specifications of the personal computer used in this research are sufficient to deal with the recursive computations linked to the SRV functions. The weight-adjusting functions and SRV functions are realized by programming C-language code in LabVIEW’s built-in Math Script tool. Other necessary blocks are selected from the function palette. This application’s front-end functions as a Graphical User Interface (GUI) to display and record real-time changes in states and control input. The software acquires the real-time state error variations to execute the control algorithm that recomputes the LQR gains to generate the updated control signal. The successive modifications in the LQR gains are scheduled after every sampling interval by using the real-time clock of the embedded processor. The SRP setup’s standard motor driver receives and modulates the updated control signals to actuate the servo motor. The durable design of the standard motor driver enables it to safely handle the system’s discontinuous control requirements.

The following physical limitations are taken into consideration while performing experiments on the Quanser SRP platform:

- Rod’s angular displacement limit: |*e*_*θ*_(*t*)| < 0.07 rad.
- Arm’s angular displacement limit: |*e*_*α*_(*t*)| < 2.97 rad.
- Control input limit: |*V*_*m*_(*t*)| < 20.0 V.

These restrictions are determined empirically. Due to the mechanics of the pendulum, the rod collapses if *e*_*θ*_ exceeds the range specified above. Similarly, the data cable of the rod’s rotary encoder blocks the arm’s ability to rotate if *e*_*α*_ exceeds the range specified above, causing the rod to collapse. To prevent the motor’s winding from overheating or needless wear and tear, the control input is maintained within ±20.0 V. Every experimental trial starts with the manual erection and stabilization of the pendulum rod. Every trial is begun with roughly the same initial conditions to ensure a fair comparative assessment of the experimental results.

### 4.2. Hardware-in-loop experiments

To validate the control performance and resilience, each control scheme is tasked with accurately regulating the pendulum rod’s vertical position and the arm’s reference position while effectively rejecting the effects of parametric variations or bounded perturbations. These controllers’ performance is analyzed via the following five customized HIL experiments. The purpose of choosing these specific experimental cases is also highlighted.

**Fig 7:**
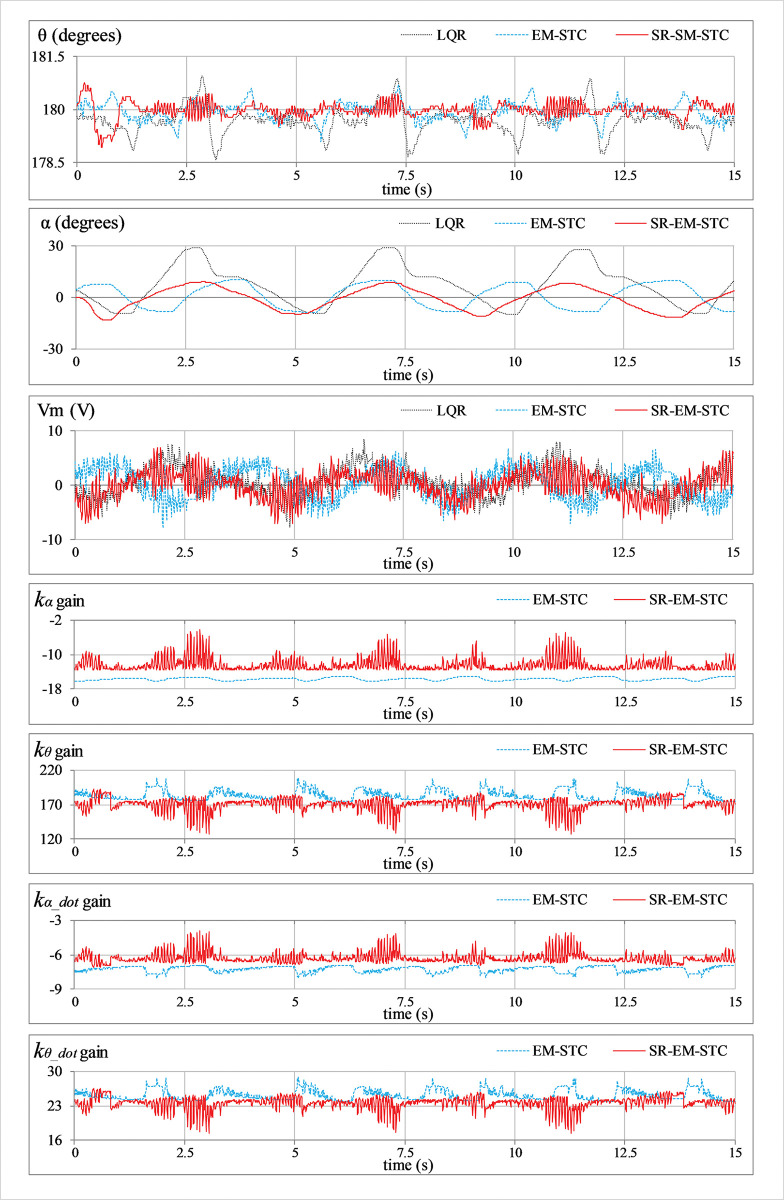
Position regulation response of the SRP under normal conditions.

**Fig 8:**
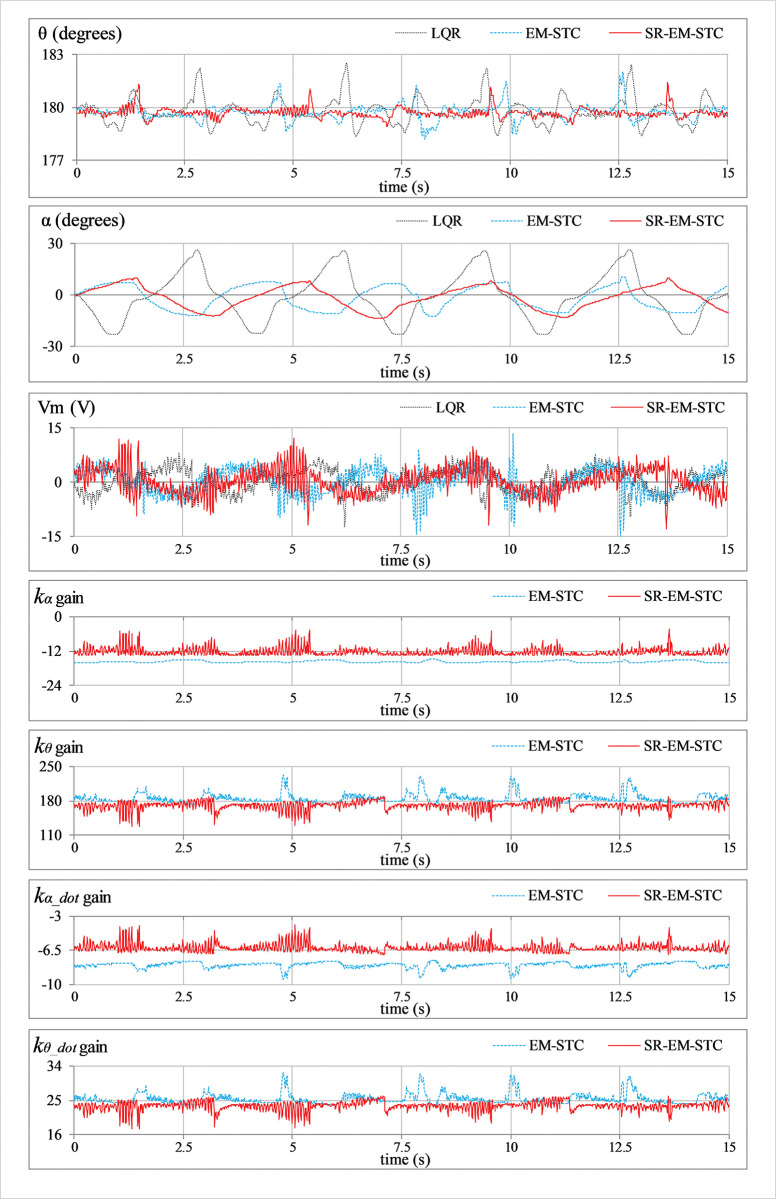
Impulse disturbance rejection behavior of the SRP.

**Fig 9:**
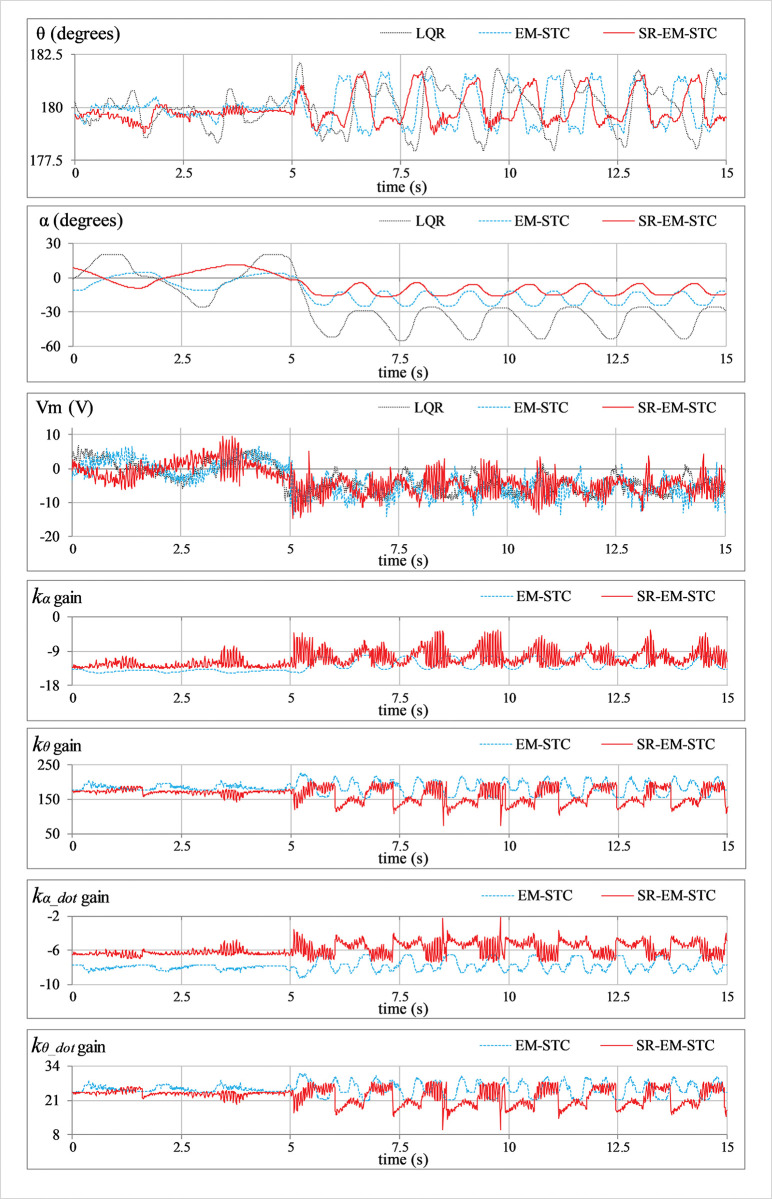
Step disturbance rejection behavior of the SRP.

**Fig 10:**
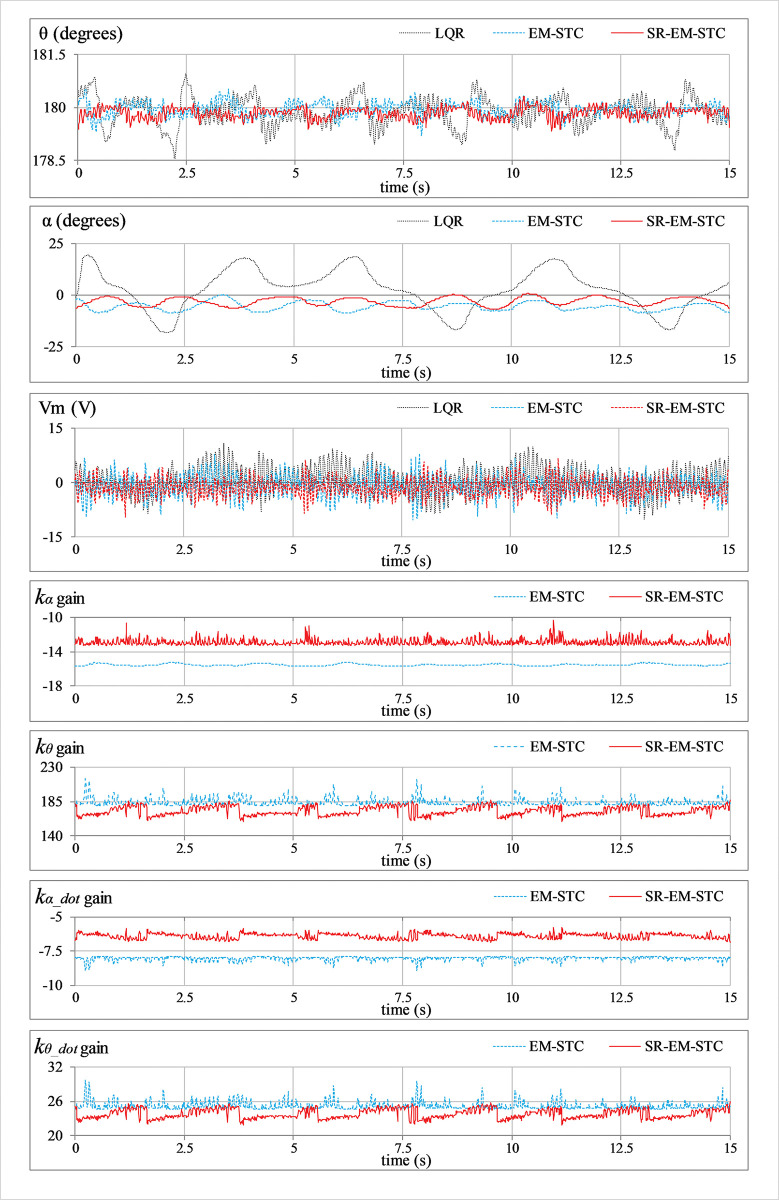
Sinusoidal disturbance attenuation behavior of the SRP.

**Fig 11:**
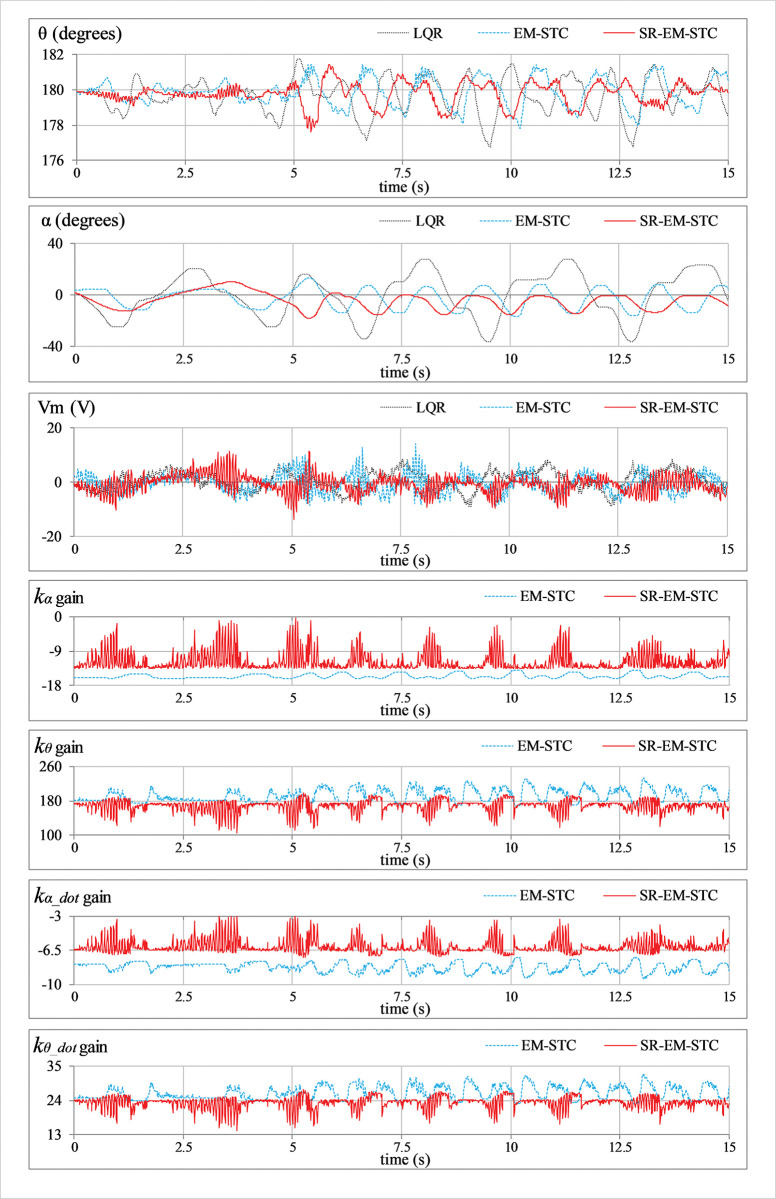
Model error compensation behavior of the SRP.

- ***Position regulation*:** This pilot test scenario is used to assess the rod’s capacity to regulate its vertical position and the arm’s ability to maintain station under normal conditions. In this test, the hardware is not subjected to any external disturbance. Fig 7 displays the consequent behavior of *θ*(*t*), *α*(*t*), *V*_*m*_(*t*), and *K*(*t*).
- ***Impulse disturbance compensation*:** By injecting a simulated impulsive signal into the control input, each controller’s capacity to reject external disturbances is analyzed. The consequent abrupt (and large) changes contributed by the impulse disturbance can potentially destabilize the system. This test case examines the physical system’s ability to quickly recover from the impact of such perturbations brought on by externally applying an impulse of a Newtonian force to the system’s body. These exogenous disturbances are generally caused by environmental uncertainties, such as transients and fluctuations in power supplies, sudden failure of hardware components, or external forces applied by seismic activity, etc. Every time the arm reaches its local maximum position, a simulated pulse with an absolute peak of 5.0 V and a temporal length of 100.0 ms is applied to disturb the response. The corresponding variations in *θ*(*t*), *α*(*t*), *V*_*m*_(*t*), and *K*(*t*) are illustrated in Fig 8.
- ***Step disturbance compensation*:** This test case examines the impact of a step perturbation (in the system’s input) on the pendulum’s ability to regulate its respective position. The aforementioned scenario emulates the application of a sudden yet constant external torque (or force) on the system, such as wind gusts and turbulence on aircraft or tidal force on a marine vessel, etc. The proposed testing aids in the assessment of transient response properties that are critical to system performance. The response is disturbed by injecting a step signal of -5.0 V into the control input signal at t = 10 sec. The corresponding variations in *θ*(*t*), *α*(*t*), *V*_*m*_(*t*), and *K*(*t*) are illustrated in Fig 9.
- ***Sinusoidal disturbance attenuation*:** This test case characterizes the immunity of the proposed control schemes against the additive noise and lumped disturbances that are normally contributed by the sensor noise, air resistance, mechanical vibrations, chattering caused by the circuit’s parasitic impedances, cogging, motor-gear backlash, friction, etc. Such disturbances are unavoidable and, thus, are widely encountered by the systems in real-world scenarios. The lumped disturbance testing aids in evaluating the robustness of the control system against exogenous noise and its capacity to filter out undesired state variations. A high-frequency, low-amplitude sinusoidal signal of this form d(t) = 1.5 sin(20πt) is introduced into the control input to perform the test. The corresponding variations in *θ*(*t*), *α*(*t*), *V*_*m*_(*t*), and *K*(*t*) are shown in Fig 10.
- ***Model error rejection*:** This test scenario mimics the occurrence of parametric variations and model identification errors in real-world engineering systems. The proposed testing scenario helps determine how well the control system can handle the discrepancies between the simulated model and the real system. This situation arises when the mathematical model used for control does not match the actual system dynamics due to real-time changes in the system’s body, such as the reduction in an aircraft’s mass during the flight due to constant fuel consumption. By connecting a mass of 0.1 kg beneath the pendulum rod, as shown in Fig 5, the adaptability of the designed controllers to modeling errors is evaluated. This mechanical alteration creates a difference between the real and reference state-space models of the system, which inevitably perturbs the system’s response. The corresponding perturbations in the time-domain profile of *θ*(*t*), *α*(*t*), *V*_*m*_(*t*), and *K*(*t*) are presented in Fig 11.

The experimental results validate the superior position-regulation and station-keeping behavior, robust disturbance-rejection capability, fast transient recovery response, and the lowest control energy consumption of the proposed SR-EM-STC. The comparative assessment of the experimental results is presented as follows.

### 4.3. Analytical discussions

The experimental outcomes of the aforementioned experiments are assessed in terms of the seven Critical Performance Indicators (CPIs) listed in Table 2.

**Table 2:** Critical performance indicators.

Symbol	Units	Description
*e*_*x_*RMS_	deg.	Root-mean-squared value of *e*_*α*_(*t*) and *e*_*θ*_(*t*).
MSV_m_	V^2^	Mean-squared value of *V*_*m*_(*t*). It indicates average control energy.
|M_p,θ_|	deg.	The absolute peak value of the overshoot in *θ*(*t*) after disturbance.
*t*_s,*θ*_ (s)	s.	Time duration of the rod’s recovery following an impulsive disturbance.
*α*_off_	deg.	Arm position offset following step disturbance.
*α*_p-p_	deg.	Peak-to-peak oscillation amplitude in the arm following the step disturbance.
V_p_	V	The absolute peak value of overshoot in control voltage following disturbance.

The quantitative analysis of each controller’s performance, as per the aforementioned CPI’s, under the influence of the testing scenarios A to E is summarized in Table 3. A concise qualitative examination of the experimental outcomes is discussed below.

**Table 3:** Quantitative comparison of experimental outcomes.

Experiments	CPI		Control Schemes		
	Symbol	Units	LQR	EM-STC	SR-EM-STC
A	*e*_*θ_*RMS_	deg.	0.48	0.27	0.23
	*e*_*α_*RMS_	deg.	14.64	6.95	6.62
	MSV_m_	V^2^	8.17	7.96	6.83
B	*e*_*θ_*RMS_	deg.	0.76	0.49	0.41
	|M_p,θ_|	deg.	2.53	2.00	1.45
	*t*_s,*θ*_	s.	0.67	0.42	0.30
	*e*_*α_*RMS_	deg.	13.85	7.04	6.74
	MSV_m_	V^2^	12.14	12.53	11.58
	V_p_	V	-13.11	-14.91	-12.78
C	*e*_*θ_*RMS_	deg.	0.98	0.85	0.70
	*e*_*α_*RMS_	deg.	32.51	16.38	10.67
	*α*_off_	deg.	-39.00	-17.75	-9.49
	*α*_p-p_	deg.	28.5	13.04	12.48
	MSV_m_	V^2^	25.86	31.81	25.13
	V_p_	V	-10.65	-14.01	-13.87
D	*e*_*θ_*RMS_	deg.	0.42	0.24	0.22
	*e*_*α_*RMS_	deg.	10.03	5.88	3.68
	MSV_m_	V^2^	13.17	9.06	7.74
E	*e*_*θ_*RMS_	deg.	1.07	0.77	0.64
	*e*_*α_*RMS_	deg.	17.25	8.24	7.85
	MSV_m_	V^2^	11.93	11.74	10.76

In **Experiment A**, the fixed-gain LQR underperforms in every aspect by demonstrating the highest *e*_*θ_*RMS_ and *e*_*α_*RMS_ while expending relatively more control energy than the other two controller variants. The EM-STC demonstrates significant improvement in attenuating position-regulation errors while cutting down on control costs. The proposed SR-EM-STC exhibits significantly better reference-tracking and station-keeping performance while further improving the control input economy.

In **Experiment B**, the LQR manifests the poorest disturbance-rejection capability, which results in slow transient recovery speed, the largest overshoots in the rod’s response, and also imposes large control input demands on the DC servo motor. The EM-STC improves the controller’s disturbance compensation ability by effectively attenuating the overshoots with a faster response speed. However, the improved robustness of EM-STC comes at the expense of significant servo control demands and extremely disrupted control activity. The proposed SR-EM-STC enables the system to exhibit relatively faster transits to the reference position and tighter damping control to reject the overshoots (or undershoots). It also suppresses the peak control input demands of the motor, which economizes control behaviour significantly.

In **Experiment C**, the LQR yields insufficient control resources to compensate for the step disturbance, which leads to a large *α*_off_ and large fluctuations in the arm’s and rod’s angular positions. The EM-STC shows a considerable improvement in effectively compensating for the step disturbance by minimizing the offset and magnitude of peak-to-peak state-error fluctuations (in the angular positions of the rod and the arm) at the expense of highly disrupted control energy expenditure. The proposed SR-EM-STC demonstrates relatively stronger disturbance rejection behavior by contributing relatively faster transient recovery, stronger attenuation against the ensuing fluctuations, and smoother control activity with relatively smaller peak actuating-torque requirements.

In **Experiment D**, the LQR exhibits the highest susceptibility to the sinusoidal disturbance, resulting in large state fluctuations and the poorest control input economy. The EM-STC demonstrates considerably better immunity against sinusoidal disturbances. However, the SR-EM-STC surpasses the aforesaid control schemes by effectively attenuating the state fluctuations and suppressing the chattering content in the rod’s position while maintaining a smooth and inexpensive overall control activity.

In **Experiment E**, the LQR manifests a subpar performance by taking mediocre actions against the modeling error, which results in large perturbations in the pendulum’s rod and the arm. The EM-STC significantly improves position-regulation behavior while slightly improving control energy consumption as well. Finally, the proposed SR-EM-STC shows the best model error compensation behavior by yielding strong damping against perturbations with relatively improved control efficiency.

In contrast to the fixed-gain LQR, the SR-EM-STC contributes an improvement of 52.2%, 54.8%, 16.4%, 55.2%, and 42.7% in the pendulum’s position regulation, arm’s position regulation, control energy expenditure, transient recovery duration, and peak overshoot magnitude, respectively. As compared to the EM-STC, the SR-EM-STC contributes a reduction of 14.8%, 4.7%, 14.4%, 28.6%, and 27.5% in the pendulum’s position regulation, arm’s position regulation, control energy expenditure, transient recovery duration, and peak overshoot magnitude, respectively. This quantitative comparison validates the enhanced time optimality of the self-regulating adaptive LQR, even under the influence of parametric uncertainties. In each experimental test case, the proposed self-regulating adaptive LQR exhibits considerable improvement in robustness as compared to adaptive LQR with fixed variation rates.

The aforementioned enhancements in the adaptive controller’s performance are indeed attributed to the self-regulating variation rates of the weight-adjusting function, which improve the DOFs of the adaptation scheme. The enhanced adaptability constructively influences the adaptation scheme’s self-reasoning capability and enables it to flexibly restructure the control scheme online to yield time-optimal and energy-efficient control decisions. The said flexibility is evident from the LQR gain variations that are depicted in the graphical illustrations of the experimental results (see Figs 7–11). In each test case, the gains contributed by the SR-EM-STC scheme demonstrate small but abrupt variations as compared to their EM-STC counterparts, which generally exhibit smoother variations. These rapid changes in the gains justify the superiority of the proposed self-regulating adaptation scheme in terms of responsiveness to parametric variations, which allows the control procedure to quickly realize and then accurately modify the critical weights to address the prevailing disturbance condition effectively.

The proposed control scheme is highly scalable and can be appropriately modified and extended to the distributed control of multiple robotic systems or networked Lagrangian systems [31, 32]. However, the scheme requires the multi-agent system’s mathematical model as well as the hyper-parameters associated with the adaptation law to be available a priori.

## 5. Conclusion

In this article, the efficacy of an adaptive LQR employing self-regulating nonlinear scaling techniques has been successfully validated for under-actuated electro-mechanical systems. The nonlinear hyperbolic scaling function with self-regulating variation rates substantially enhances the controller’s adaptability and, thus, its robustness and response speed against bounded external perturbations. The proposed self-regulation algorithm uses error dynamics in conjunction with its superior self-regulation capacity to dynamically adjust the variation rates of the weight-adjusting nonlinear functions. The self-regulating variances adaptively modify the structure of the aforementioned functions to remove any inaccuracies in their heuristic calibration. The proposed scheme exploits the full potential and harnesses the maximum flexibility of the state-error-driven HSFs. The experimental results verify that the dynamic adjustment of variances in the weight-adjusting functions enhances the controller’s effectiveness in compensating for the nonlinear complexities and parametric variations encountered by real-world systems. The proposed scheme increases the transient response speed, amplifies the control stiffness against the reference-tracking fluctuations, and minimizes the peak magnitudes in the actuator’s control profile while maintaining the stability of the controller under every operating condition. In the future, this scheme can be examined by extending it to other under-actuated mechatronic systems for further validation of its design scalability. Other online self-organizing algorithms can also be investigated for the adjustment of shape variations in real time.
